# The mediating role of incentives in association between leadership attention and self-perceived continuous improvement in infection prevention and control among medical staff: A cross-sectional survey

**DOI:** 10.3389/fpubh.2023.984847

**Published:** 2023-02-09

**Authors:** Lu Wang, Dandan Zhang, Junjie Liu, Yuqing Tang, Qian Zhou, Xiaoquan Lai, Feiyang Zheng, Qianning Wang, Xinping Zhang, Jing Cheng

**Affiliations:** ^1^School of Medicine and Health Management, Tongji Medical College, Huazhong University of Science and Technology, Wuhan, China; ^2^The First Affiliated Hospital, Nanjing Medical University, Human Resource Office, Nanjing, China; ^3^School of Statistics and Mathematics, Central University of Finance and Economics, Beijing, China; ^4^Department of Nosocomial Infection, Tongji Hospital, Tongji Medical College, Huazhong University of Science and Technology, Wuhan, China; ^5^Department of Emergency, Tongji Hospital, Tongji Medical College, Huazhong University of Science and Technology, Wuhan, China

**Keywords:** infection control, leadership, continuous improvement, incentive, mediating analysis, medical staff

## Abstract

**Objectives:**

Promoting improvement in Infection Prevention and Control (IPC) is an important part of improving the quality of care. The influence of leadership attention and incentives on the self-perceived continuous improvement in IPC has drawn a lot of attention, but relevant academic research is still lacking. The objective of this study is to explore the effect of leadership attention on self-perceived continuous improvement in IPC among medical staff and its underlying mechanisms.

**Method:**

The 3,512 medical staff from 239 health facilities in Hubei, China, were surveyed online during September 2020. Data on leadership attention, incentives, and improvement in Infection Prevention and Control were collected using self-administered questionnaires. Correlation analysis was used to analyze the relationship between leadership attention, incentives, and improvement in Infection Prevention and Control. Amos 24.0 was used to analyze the mediating role.

**Results:**

The scores of leadership attention, incentives and self-perceived continuous improvement in Infection Prevention and Control were all high. The score of leadership attention was the highest (4.67 ± 0.59), followed by self-perceived continuous improvement (4.62 ± 0.59) and incentives in Infection Prevention and Control (4.12 ± 0.83). Leadership attention positively affected self-perceived continuous improvement in Infection Prevention and Control (β = 0.85, 95% CI = [0.83, 0.87]). Moreover, incentives partially mediated the effect of leadership attention on self-perceived continuous improvement in Infection Prevention and Control among medical staff (β = 0.13, 95% CI = [0.12, 0.15]).

**Conclusion:**

Leadership attention positively affects self-perceived continuous improvement in Infection Prevention and Control among medical staff, and incentives mediates this relationship. The present study has valuable implications for self-perceived continuous improvement in Infection Prevention and Control from the perspective of leadership attention and incentives.

## Introduction

Healthcare-associated infections (HAIs) have become a major global threat to health systems. 1.7 million people acquire HAIs in the United States annually, resulting in more than 98,000 deaths (1). More than 2.6 million new cases of nosocomial infection occur every year in Europe, with a cumulative burden estimated in disability-adjusted-life-years higher than most infectious diseases (1). Continuous improvement in IPC is a longstanding problem within healthcare systems in many countries (2–8). The 2019 novel coronavirus disease (COVID-19), in particular, exposed many IPC quality improvement issues that need to be urgently addressed. The organizational management system and structure of nosocomial infection need to be improved, and the allocation and training of professional staff is also a severe problem (9–12).

Continuous improvement in IPC involves improving operations, system processes, working environment, or regulatory compliance, focusing on the process improvement of the organization, which reflects the improvement culture and climate of the organization (13). The positive response to HAIs can promote the continuous improvement of the organizational system. Therefore, whether the organization can make continuous improvements depends on the learning ability of the organization. And the excellent operation of an organization is inseparable from the mechanism of continuous improvement (14). The improvement in IPC at the organizational level is crucial for the successful prevention of HAIs, including the outbreak of COVID-19, through high-quality care within the context of universal health coverage (4). However, IPC execution was poor, and the status of IPC improvement was not optimistic (15). According to previous studies, the compliance rate of medical staff toward IPC guidelines was <50% (16), and the implementation rate of hand hygiene with the highest prevalence was only 40% overall (9). Therefore, more attention should be paid to the improvement in IPC, especially to the influencing factors and the formation mechanism of the improvement in IPC, to develop targeted measures to enhance IPC quality.

The continuous improvement in IPC can be influenced by both patient and medical factors. From the perspective of the medical, individual and organizational factors affect continuous improvement in IPC (17). It is considered that strengthening leadership attention and incentive systems as important organizational management principles for IPC improvement (18). Another study also indicated that the clinical application of continuous improvement projects had a pervasive impact when it took place within organizational leadership, such as a supportive regulatory environment aligned with financial incentives (19). However, how leadership attention affects the continuous improvement in IPC should be further explored. Some studies pointed out that incentives may be an important mediating variable to explain how leadership attention affects the continuous improvement in IPC (20–22). The incentive model by Porter and Lawler assumes that behavior and incentives are inseparable, and incentives mediate the relationship between leadership attention and continuous improvement (23). The mechanism of Porter and Lawler's incentive model could thus be interpreted as that leadership attention enhances incentives, thereby improving continuous improvement (24–26).

This study intends to explore the relationship between leadership attention and self-perceived continuous improvement in IPC, and its internal mechanism, focusing on the mediating role of incentives. This study has significant enlightenment for leadership construction and incentive system construction in the IPC field, thus providing an evidence-based suggestions for the comprehensive management and continuous improvement in IPC at the organizational level. So far, this study is the first to quantitatively describe the level and distribution of leadership attention, incentives, and self-perceived continuous improvement in IPC and to elaborate the mechanism of leadership attention on self-perceived continuous improvement in IPC with a large sample size.

## Literature review and hypotheses development

### Leadership attention and continuous improvement in IPC

Leadership attention in IPC means the commitment that leaders give to HAIs (27), which was mainly reflected in leadership behaviors, such as leaders support and participation in work in IPC (28–30), leaders supervision of work in IPC (31), and leaders attention to opinions on improving work in IPC (32), etc. Leadership attention, an essential part of leadership, is the key to strengthening the continuous improvement of the health system (33). The path-goal theory suggests four leadership styles: directive, supportive, participative, and achievement-oriented (34). Leaders begin by assessing the characteristics of their subordinates and any environmental factors, then choose the leadership style most suited to their assessment. After that, they will focus on the key motivational factors to ensure their subordinates are motivated to hit their objectives, ultimately achieving continuous improvement of the organization (34). Many researchers emphasized the importance of early leadership participation and support for continuous improvement efforts (33, 35, 36). Weiner et al. (30) pointed out that senior leaders' participation in clinical continuous improvement projects could help increase the effect of continuous improvement. Vaughn et al. (36) insisted that if leaders spent more than 25% of their time on the management of continuous improvement, they would acquire a better quality index score. Management's commitment to safety will positively impact safety management and behaviors (37). Consequently, we formulated the following hypothesis:

H1: Leadership attention positively affects the continuous improvement in IPC.

### Leadership attention and incentives

The incentive is a process in which personal demands are created under the action of external or internal stimuli, motives and behaviors are generated under the guidance of demands, and finally, some goals are achieved (38). Incentive in IPC means that moral or material rewards were adopted to encourage medical staff to achieve organizational and personal goals and produce high-quality performance effectively (39). Leadership attention is closely related to incentives. According to the path-goal theory, leaders should adopt different leadership styles and incentive measures according to various subordinates and environmental characteristics (34). Incentive measures were more likely to be taken when leaders emphasized nosocomial infection (40). Managers with greater career ambition and task attention would respond more to incentive compensation (41). Another study showed that the leaders who focused on strengthening leadership attention and improving leadership behavior were most likely to increase incentives (42). Bettinger et al. (43) and Islam et al. (44) mentioned that leadership attention might positively affect incentives. Thus, it can be assumed that:

H2: Leadership attention positively affects incentives in IPC.

### Incentives and continuous improvement in IPC

Incentives are important factors affecting continuous improvement. The incentive model by Porter and Lawler (23) proposed that effective continuous improvement can be promoted by creating extrinsic and intrinsic rewards. Effective incentives, such as establishing reasonable systems and standards of rewards and punishments, could motivate medical staff to attach importance to nosocomial infection and improve patient outcomes (45, 46). The use of incentive components and strategic alignment of quality goals with physician financial incentives in intensive care can positively affect the quality of care by physicians (47). Continuous improvement of safety performance could be promoted by punishment for dangerous behavior in the field of transportation (48). Incentive programs are being increasingly utilized in the realm of health care to change patient behaviors and promote continuous improvement of health outcomes (22, 49). These programs can range from one-time incentives for preventative care to long-term incentives for goals such as smoking cessation (50), blood donation (51), and so on. Consequently, incentives in IPC may be an essential factor in promoting continuous improvement in IPC. The hypothesis is as follows:

H3: Incentives positively affect the continuous improvement in IPC.

### Leadership attention, incentives, and continuous improvement in IPC

Incentives may play a mediating role in the relationship between leadership attention and continuous improvement. The path-goal theory illustrates that leaders could help their subordinates to achieve their goals through incentive mechanisms, with incentives as an intermediary role (34). A study found that leadership attention positively affected the performance of pharmaceutical workers through motivation, confirming the mediating role of motivation by surveying 220 pharmacists in Vietnam (20). And incentives positively affect motivation (43), so we can assume that incentives may mediate the relationship between leadership attention and the continuous improvement in IPC. Accordingly, the following hypothesis could be put forward:

H4: Leadership attention positively affects the continuous improvement in IPC mediated by incentives.

**Figure 1:**
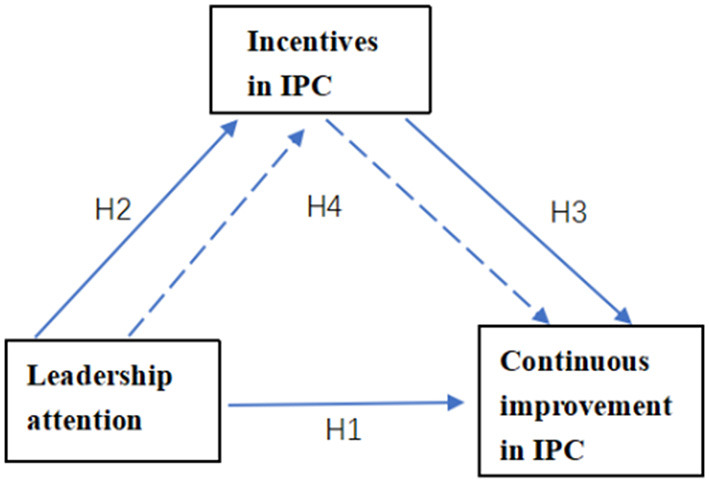
The theoretical hypothesis model.

The four research hypotheses are summarized in the model (Figure 1).

## Methods

### Design, setting and participants

An online survey was conducted among medical staff from all 237 comprehensive medical institutions who participated in the Training course on hospital infection management (the content of the Training course on hospital infection management does not involve the relevant content of the questionnaire in this study), in September 2020, Hubei Province, China, through a stratified random sampling method.

Based on institutional stratification, we encouraged each comprehensive medical institution to randomly select medical staff from different departments to attend the conference. In order to ensure the number of questionnaires, participants were encouraged to send the questionnaire link to their colleagues who were engaged in nosocomial infection but could not attend. Each comprehensive medical institution was required to provide at least 17 questionnaires. Hubei, China was chosen as the research site because coronavirus disease 19 (COVID-19) originated in Hubei, and developed into a major public health issue in China. It has a dense population, a large number of medical institutions, and rich medical and health resources. Inclusion criteria: (1) doctors or nurses working in clinical departments; (2) engaged in nosocomial infection prevention and control; and (3) informed and voluntary participation in the study. Exclusion criteria: management personnel.

### Data collection procedure

The survey was conducted from September 17 to September 19, 2020. We invited all comprehensive medical institutions in Hubei province to participate in the Training course on hospital infection management in the form of online learning. The survey was conducted before the start of the training course. Before the investigation, the link to the questionnaire was posted on the home page of the live broadcast platform. Then, the purpose and content of the survey were explained to respondents through an online medium. It was emphasized that the study was to improve the status quo of IPC management in hospitals. Each respondent was required to fill in the questionnaire according to their actual condition. Whether to fill in the questionnaire is voluntary and will not affect the subsequent study. The online meeting lasted for 3 days, and the data was exported and analyzed before 24:00 every day.

To ensure the quality of data, we adopted the double-check method according to the following requirements: All items were set as “required questions” to guarantee the completeness of the questionnaire. Users from each IP address only had one chance to participate in the investigation. The answer time was longer than 1 min.

### Measurements

The questionnaire was developed based on the tools of leadership attention, incentives and improvement in the field of hospitals safety, and the characteristics of the IPC field. The self-report questionnaire consisted of four parts: demographic characteristics, leadership attention, incentives, and self-perceived continuous improvement in IPC. Demographic characteristics were collected, including clinical department, gender, age, professional title, working years, title, education level, etc. The measurement variables included Leadership attention, Incentives in IPC, and self-perceived continuous improvement in IPC. Items were scored on a five-point Likert Scale from 1 “strongly disagree” to 5 “strongly agree.” The higher the score, the more leadership attention and the better incentives and self-perceived continuous improvement in IPC.

### Leadership attention

Leadership attention was measured with three items adapted from Patient Safety Climate in Healthcare Organizations (PSCHO) (52) and the concept of leadership attention (28, 31, 32). PSCHO was an instrument for assessing patient safety culture with good reliability and validity. The dimension of senior managers' engagement and the overall emphasis on safety at the organizational level was referred to in this study. Item 1 was derived from PSCHO, and items 2 and 3 were adapted from the concept of leadership attention. The final items were as follows: a1. Leaders in my unit support and actively participate in IPC tasks. a2. Leaders in my unit supervise and check IPC tasks at any time. a3. Leaders in my unit value suggestions on improving IPC work. The Cronbach's alpha was 0.92.

### Incentives in IPC

Incentives in IPC was measured with four items adapted from PSCHO (49) and the concept of incentives (39, 40). Items from the dimension of Unit recognition and support for safety in PSCHO were adopted, such as I am rewarded for taking quick action to identify a severe mistake; If people find out that I make a mistake, I will be disciplined; My unit recognizes individual safety achievement through rewards and incentives. With the concept of incentives, the final items are a4. Evaluation results in IPC will affect the income of staff in our unit. a5. Staff in my unit will be praised for pointing out hidden dangers or unsafe behaviors. a6. Staff in my unit will receive material rewards for better IPC performance. a7. Apart from the material rewards, staff in my unit will also get other rewards (e.g., promotion) for better IPC performance. The Cronbach's alpha was 0.82.

### Self-perceived continuous improvement in IPC

After a HAI incident occurs, whether the organization can learn from it and optimize and improve it in an orderly manner is crucial to avoid the recurrence of similar hospital infection incidents (14). Feedback can improve employee engagement and quality of continuous improvement, so employee feedback is an integral part of IPC continuous improvement (53). Our study focused on measuring self-perceived continuous improvement of the medical staff for the organization, which to some extent reflected the organization's ability to learn and a culture of continuous improvement.

Self-perceived continuous improvement in IPC was measured with four items adapted from Hospital Survey on Patient Safety Culture (HSOPSC) (54) and concept of self-perceived continuous improvement in IPC (15). Items from the dimension of Organizational learning–continuous improvement in HSOPSC was adopted, such as we are actively doing things to improve patient safety; mistakes have led to positive changes here; after we make changes to improve patient safety, then we evaluated their effectiveness. With the concept of self-perceived continuous improvement in IPC, the final items are a8. My unit will regularly assess workflow to determine whether it needs improvement. a9. My unit will evaluate timely IPC measures to see how well they worked. a10. My unit will adjust workflow timely to ensure that the same HAIs will not happen again. a11. Staff in my unit will regularly receive feedback on the effect of continuous improvement. The Cronbach's alpha was 0.96.

### Reliability and validity

The overall Kaiser–Meyer–Olkin (KMO) value of the questionnaire was 0.932. The Bartlett sphericity test was significant (χ^2^ = 38,655.00, df = 55, *P* < 0.000), suitable for factor analysis. The KMO value of leadership attention, incentives, and self-perceived continuous improvement in IPC was >0.6 and *P* < 0.05, indicating that the data met the requirement of factor analysis. The factor loading coefficient of each item was >0.6, which showed that the extracted common factors are highly correlated. When using real-world data to verify and evaluate the theoretical model, χ^2^ is susceptible to sample size. The larger the sample size, the more significant the results, and the easier to reject the theoretical model (55). The results of confirmatory factor analysis (CFA) showed that all the fitting indexes meet the requirements except for χ^2^/*df* [Root Mean Square Error of Approximation (RMSEA) = 0.064, Root Meansquare Residual (RMR) = 0.03, Normed Fit Index (NFI) = 0.99, Relative Fitting Index (RFI) = 0.98, Comparative Fit Index (CFI) = 0.99, Incremental Fit Index (IFI) = 0.99, Tucker–Lewis Fit Index (TLI) = 0.98, Parsimonious Goodness Fit Index (PGFI) = 0.56, Parsimonious Normed Fit Index (PNFI) = 0.68, Parsimonious Comparative Fit Index (PCFI) = 0.68], which indicated the structure validity is good.

Convergent validity refers to the convergent degree of multiple observation variables on the same construct measured by different methods (56). The factor load is generally required to be >0.5, and the combination reliability is required to be >0.7; The Average Variance Extracted (AVE) is needed to be >0.5. In this study, the factor load coefficients all meet the requirements. The combined reliability of leadership attention, incentives, and self-perceived continuous improvement in IPC was 0.92, 0.96, and 0.84, respectively. AVE values were all >0.5. The absolute values of dimension correlation coefficients of all variables were <0.5 and less than the square root of AVE, indicating that the questionnaire has good convergence validity and discrimination validity.

We adopted the graded response model (GRM) of Item Response Theory (IRT) to assess the differentiation, difficulty, information function and *S*-*X*^2^ (Item Fit Index) (57) of the questionnaires in this study. The results showed that the differentiation ranged from 1.63 to 28.45, with an average value of 7.83. The difficulty level increases monotonically (−2.70 to 0.19), with a reasonable range of values, and all *S*-*X*^2^ were up to standard (*P* <0.05). Therefore, the questionnaire adopted in our study has good differentiation, appropriate difficulty and reasonable options.

### Statistical analysis

Descriptive statistics and Pearson's correlations were calculated using SPSS 26.0 for leadership attention, incentives, and self-perceived continuous improvement in IPC. Amos 24.0 was used to establish a structural equation model between leadership attention, incentives, and self-perceived continuous improvement in IPC to test the mediating effect of incentives in the relationship between leadership attention and self-perceived continuous improvement in IPC. Path coefficients were used to examine the action paths of leadership attention on improvement (including direct and indirect effects). The Bootstrap method was adopted to test the mediation effect. The sample size was selected as 5,000. Under the 95% confidence interval, if the result of the mediation test contained 0, it indicated that the mediation effect of IPC incentives was not significant; if the result of the mediation test did not contain 0, it suggested that the mediation effect of IPC incentives was significant. In this study, *p* < 0.05 was considered to be statistically significant.

### Ethical procedures

This study was approved by the Ethics Committee of Tongji Medical College, Huazhong University of Science and Technology (No: 2020S252). All participants were enrolled in the investigation using the principles of informed consent and confidentiality.

## Result

### Common method variance

Common method variance (CMV) is the systematic error variance shared among variables measured with the same source or method. This systematic error variance could also bias the estimated relationships among variables or measures (58). To test for any potential CMV, Harman's single factor test was conducted with CFA, where all items were loaded onto a common factor, and the model exhibited poor fit (χ^2^/*df* = 134.04, RMSEA = 0.20, RMR = 0.11, NFI = 0.85, RFI = 0.81, CFI = 0.85, IFI = 0.85, TLI = 0.81), which indicated there was no significant bias.

### Demographic characteristics

A total of 4,000 medical staff in clinical departments participated in the survey, 3,512 of which were remained, whereas 488 were removed because the answers to all 11 items in a questionnaire were the same or because the questionnaire was not filled out by doctors or nurses, or the answer time was <1 min. The effective response rate was 87.80%. 16.00% were male, and 84.00% were female. The average age was 30.52 ± 1.26 (mean ± SD). 25.60% were doctors, and 74.40% were nurses. 77.73% had a bachelor's degree or above. 53.22% worked for more than 5 years. 45.02% have intermediate or higher professional titles. 76.82% of them had contact with hospital-infected patients. There were no significant differences in scores of demographic variables among different groups on self-perceived continuous improvement in IPC, which indicated that demographic variables did not affect the level of IPC improvement. The specific demographic characteristics are shown in Table 1.

**Table 1:** Demographic characteristics and differences in self-perceived continuous improvement in IPC (*N* = 3,512).

**Variable**	***N* (%)**	**Self-perceived continuous improvement in IPC**		
		**Mean** ±**SD**	***t/F*** **-value**	***P*** **-value**
**Clinical departments**				
Internal medicine	1,312 (37.36%)	4.63 ± 0.60		
Surgery	801 (22.81%)	4.65 ± 0.55		
Obstetrics and gynecology	258 (7.35%)	4.53 ± 0.68	2.01	0.07
Peadiatrics	401 (11.42%)	4.62 ± 0.59		
Emergency	237 (6.75%)	4.62 ± 0.56		
Others	503 (14.32%)	4.63 ± 0.54		
**Gender**				
Male	561 (15.97%)	4.50 ± 0.64	0.08	0.78
Female	2,951 (84.03%)	4.51 ± 0.62		
**Age (years)**				
18–25	420 (11.96%)	4.54 ± 0.58		
26–35	1,788 (50.91%)	4.50 ± 0.66	1.70	0.13
36–45	808 (23.01%)	4.52 ± 0.61		
>46	491 (13.98%)	4.49 ± 0.61		
**Professional title**				
Doctor	899 (25.60%)	4.48 ± 0.61	2.44	0.12
Nurse	2,613 (74.40%)	4.52 ± 0.62		
**Working years**				
< 1	427 (12.16%)	4.63 ± 0.50		
1–3	724 (20.62%)	4.50 ± 0.64		
3–5	490 (13.95%)	4.52 ± 0.60	1.43	0.22
5–10	966 (27.51%)	4.52 ± 0.63		
>10	903 (25.71%)	4.49 ± 0.62		
**Title**				
Junior	1,931 (54.98%)	4.53 ± 0.63		
Intermediate	1,194 (34.00%)	4.49 ± 0.61	1.36	0.25
Deputy senior	335 (9.54%)	4.46 ± 0.63		
Senior	52 (1.48%)	4.46 ± 0.54		
**Education**				
Junior college or below	782 (22.27%)	4.46 ± 0.65		
bachelor degree	2,503 (71.27%)	4.52 ± 0.62	2.68	0.07
Master degree or above	227 (6.46%)	4.54 ± 0.57		
**Members of the IPC team**				
Yes	1,120 (31.89%)	4.51 ± 0.59	0.04	0.84
No	2,392 (68.11%)	4.51 ± 0.64		
**Contact with hospital-infected patients**				
Yes	2,698 (76.82%)	4.50 ± 0.62	0.97	0.38
No	814 (23.18%)	4.53 ± 0.61		

### Distribution and correlations analysis among leadership attention, incentives, and self-perceived continuous improvement in IPC

Table 2 showed that the scores of leadership attention, incentives, and self-perceived continuous improvement in IPC were high after COVID-19. The leadership attention score was the highest (4.67 ± 0.59), followed by self-perceived continuous improvement in IPC (4.62 ± 0.59) and incentives in IPC (4.12 ± 0.83). Among them, scores of all items in leadership attention and incentives in IPC were more than 4.5 points. While items of A10 “My unit timely adjust workflow to ensure that the same HAIs are not happen again” and A11 “Workers regularly receive feedback on the effect of continuous improvement” under the dimension of self-perceived continuous improvement in IPC scored relatively low (<4.0 points).

**Table 2:** Means, standard deviations, and correlations for all variables.

**Variables**	**Mean ±SD**	**1**	**2**	**3**
1. Leadership attention	4.67 ± 0.59	1		
A1	4.70 ± 0.62			
A2	4.66 ± 0.65			
A3	4.65 ± 0.65			
2. Incentives in IPC	4.12 ± 0.83	0.54^**^	1	
A4	4.65 ± 0.61			
A5	4.63 ± 0.62			
A6	4.64 ± 0.62			
A7	4.58 ± 0.67			
3.Self-perceived continuous improvement in IPC	4.62 ± 0.59	0.85^**^	0.63^**^	1
A8	4.18 ± 1.06			
A9	4.45 ± 0.79			
A10	3.89 ± 1.15			
A11	3.97 ± 1.11			

** p < 0.01.

The correlations for each factor are presented in Table 2. As hypothesized, leadership attention was positively correlated with incentive in IPC (*r* = 0.54, *p* < 0.01), and correlated with self-perceived continuous improvement in IPC (*r* = 0.85, *p* < 0.01). Incentive in IPC was positively correlated with self-perceived continuous improvement in IPC (*r* = 0.63, *p* < 0.01).

### Mediating effect of incentives in IPC

**Figure 2:**
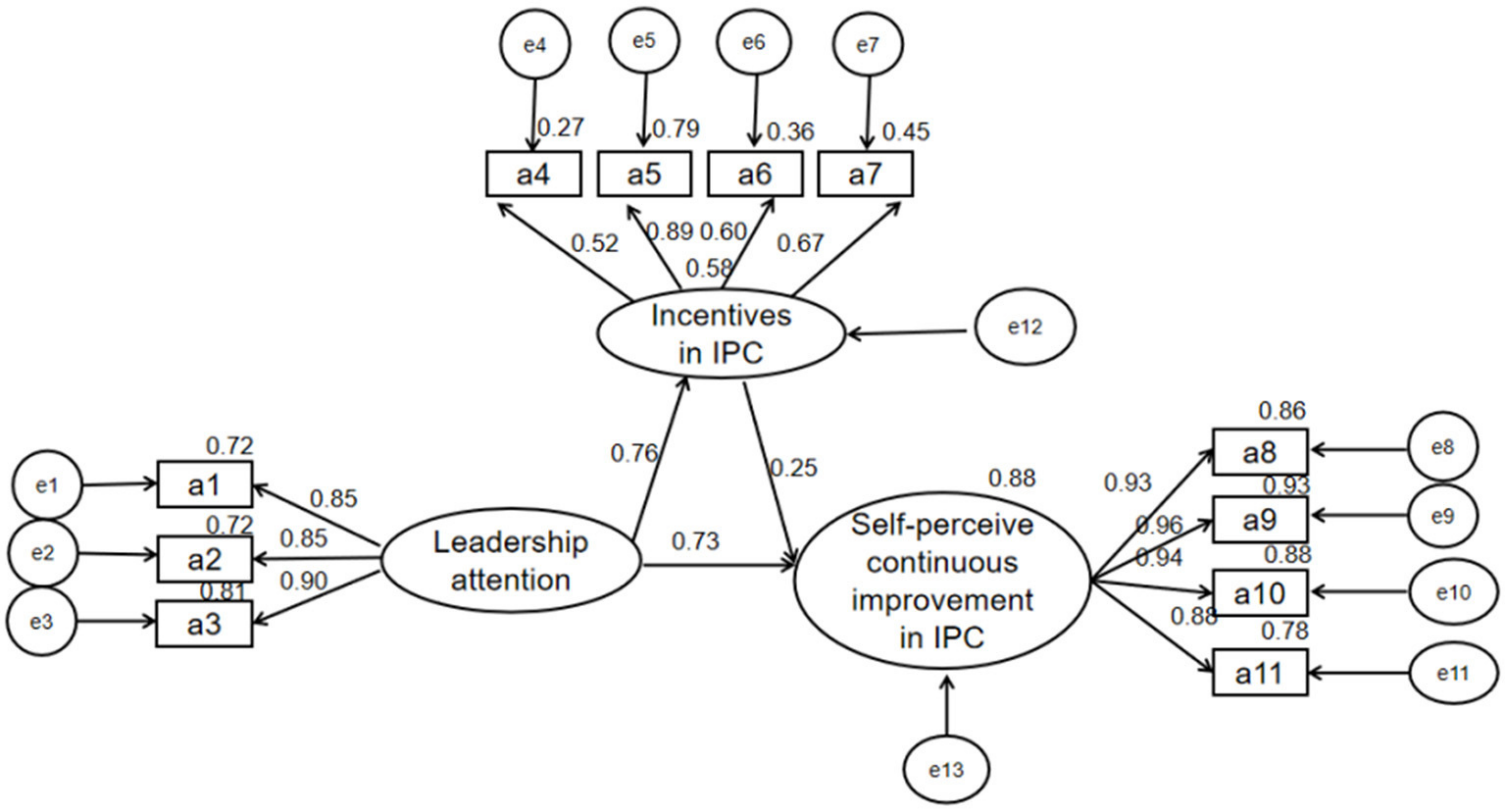
Mediating model of incentives between leadership attention and self-perceived continuous improvement in IPC.

Figure 2 showed the path diagram and the path coefficients between variables. The path coefficients between leadership attention and self-perceived continuous improvement in IPC (β = 0.73, *p* < 0.01), leadership attention and incentives in IPC (β = 0.76, *p* < 0.01), incentives in IPC and self-perceived continuous improvement in IPC (β = 0.25, *p* < 0.01) were all statistically significant.

Five thousand bootstrap tests were performed on the mediation effect, and the results showed that the path differences in the model were statistically significant (Table 3). The total effect of leadership attention on self-perceived continuous improvement in IPC was 0.85 (95%CI: 0.83–0.87, *p* < 0.01), the direct effect was 0.72 (95%CI: 0.70–0.74, *p* < 0.01), and the indirect effect was 0.13 (95%CI: 0.12–0.15, *p* < 0.01). Hence, incentives in IPC had a mediating effect between leadership attention and self-perceived continuous improvement in IPC, the mediating effect was 13%, and the mediating effect accounted for 15% of the total effect.

**Table 3:** Bootstrap test of the mediating effect of leadership attention on self-perceived continuous improvement in IPC.

**Pathways**	**β**	**Standard error**	**95%CI**	***P***	**Results**
**Total effect**					
Leadership attention → self-perceived continuous improvement in IPC	0.85	0.01	0.83–0.87	<0.01	
**Direct effect**					
Leadership attention → self-perceived continuous improvement in IPC	0.72	0.01	0.70–0.74	<0.01	H1 supported
**Indirect effect**					
Leadership attention → incentives	0.76	0.02	0.72–0.80	<0.01	H2 supported
Incentives → self-perceived continuous improvement in IPC	0.18	0.01	0.16–0.19	<0.01	H3 supported
Leadership attention → incentives → self-perceived continuous improvement in IPC.	0.13	0.01	0.12–0.15	<0.01	H4 supported

CI, confidence interval.

## Discussion

This study examined the direct (0.72), indirect (0.13), and total effects (0.85) of leadership attention on self-perceived continuous improvement in IPC mediated by incentives among medical staff. Our findings expand the existing literature by demonstrating the mediating role of incentives on the association between leadership attention and self-perceived continuous improvement in IPC.

This study constructed a tool with good reliability and validity for measuring leadership attention, incentives, and self-perceived continuous improvement in IPC. This study was the first to quantitatively describe the level and distribution of these three variables after COVID-19. The results showed that the scores of leadership attention, incentives, and self-perceived continuous improvement in IPC were high after COVID-19, and the score of leadership attention was the highest. The possible reason may be that medical institutions pay more attention to IPC work, and the management mode of full participation of medical staff in IPC in hospitals has been widely used after COVID-19 (59). The current IPC work in hospitals advocates “whole-process management.” Coordination and cooperation among departments are the basis for IPC and also the key to continuous improvement in IPC (60, 61). At the same time, we were aware that the scores of items A10 and A11 remain low, indicating that the level of IPC continuous improvement still needs to be improved. China contained the epidemic quickly, which was a test of the effect of IPC improvement, but it still exposed some problems. For examples, inadequate personal protection of medical workers in the early stages of the epidemic, personal protective equipment (PPE) shortages, and insufficient training in infectious diseases for front-line medical staff (except infectious disease physicians) (62).

Furthermore, compared with the score of leadership attention and self-perceived continuous improvement in IPC, the overall score of Incentives in IPC was relatively low, indicating that the effect of the incentive system was limited and needed continuous improvement although hospital and department managers attached great importance to IPC work. Studies showed that some hospitals still used punishment as the primary management method for IPC work in hospitals, which was repellent to medical staff and even hindered IPC work in hospitals (63). Good assessment and evaluation systems in IPC can enhance the management effect of IPC and reduce the incidence of HAIs (64). Therefore, it is necessary to focus on improving the incentive system in IPC to advance the quality of IPC work.

This study showed that leadership attention positively affected self-perceived continuous improvement in IPC (H1), which was consistent with previous studies. Nguyen et al. (20) pointed out that transformational leadership positively affected job performance in the pharmaceutical field (Direct effect: 0.11~0.41). Jiang et al. (65) concluded that transformational leadership positively affected workers' sustainable improvement in the construction industry (Direct effect: 0.00~0.19). Piccolo and Colquitt (66) and Vaughn et al. (36) also demonstrated this hypothesis. Furthermore, we observed that the direct effect of this study was higher than that of the previous research. The possible reason was that this study was conducted after COVID-19, which aroused the attention of specialists in IPC and even leaders worldwide to strengthen the continuous improvement in IPC (59). In contrast, some studies had shown insufficient leadership attention to preventing and controlling HAIs before COVID-19 due to the consideration of hospital economic benefits and cost, so the continuous improvement in IPC was limited (67–69). Therefore, leadership attention should be strengthened to improve the performance in IPC.

One of the most important findings of this study was to confirm that incentives in IPC mediated the effect of leadership attention on self-perceived continuous improvement in IPC (H4). That indicated that leadership attention not only positively affected self-perceived continuous improvement in IPC directly, but also positively affected self-perceived continuous improvement in IPC indirectly mediated by incentives in IPC. The mediating effect of incentives in IPC can be understood from the following two aspects. On the one hand, leadership attention can positively affect incentives in IPC (H2) (Direct effect: 0.76), which was similar to previous studies. Basahel (70) proved that safety leadership positively affected safety incentives (Direct effect: 0.61), which was consistent with the findings of Susanto and Nopiyanti (71) (Direct effect: 0.21) and Paarlberg and Lavigna (72) (Direct effect: 0.13–0.35). And we found that the effect value in this study was slightly higher. The reason may be that COVID-19 had attracted leadership attention worldwide, with incentives to strengthen the construction of infection infrastructure and professional teams, to contain the spread of COVID-19 in China (73–75). On the other hand, incentives in IPC can positively affect self-perceived continuous improvement in IPC (H3) (Direct effect: 0.18), which was consistent with previous studies. Pancasila et al. (76) showed that work motivation positively affected work performance (Direct effect: 0.17). Nasution (77) suggested that incentive remuneration positively affected teacher continuous improvement (Direct effect: 0.35). Fuller and Harding (78) and Hersona and Sidharta (79) also demonstrated this hypothesis. In addition, incentives such as pay-for-performance (80) or value-based reimbursement schemes (81) can also promote continuous improvement of IPC.

Based on the above, incentives in IPC mediated the relationship between leadership attention and self-perceived continuous improvement in IPC (the mediation effect: 0.13). Some studies have proved the mediating role of intrinsic motivation in leadership attention and continuous improvement. Hersona et al. (79) confirmed that transformational leadership positively affected sports continuous improvement through intrinsic motivation, confirming the mediating role of intrinsic motivation by conducting a survey on athletes and coaches in a small university. Nguyen et al. (20) and Jiang et al. (65) also proved this point. There is no empirical study on the relationship between leadership attention, incentive, and self-perceived continuous improvement in IPC. And incentives positively affect motivation (43). Therefore, incentives in IPC mediated the relationship between leadership attention and self-perceived continuous improvement in IPC. It implied that leadership attention should be strengthened, and effective incentive systems should be adopted to promote the self-perceived continuous improvement in IPC.

## Strengths and limitations

The strength of our study is that we are the first to quantitatively describe the level and distribution of leadership attention, incentives, and self-perceived continuous improvement in IPC and to elaborate on the mechanism of leadership attention on self-perceived continuous improvement in IPC from the perspective of organization management. Moreover, the sample size of our study is large, which is rarely seen in previous studies. However, there are several limitations. First, the measurement tool in this study adopts self-reported, and there may be situations where subjects overestimate or underestimate measured variables. But in the absence of objective measurement tools, subjective measurement is an alternative. Future research could try to construct more objective measurement tools. Second, the participants of this study are from one province, and this is a cross-sectional study, which may affect the generalization of this research. Future studies can further expand the scope of the survey and design a prospective cohort study to confirm our study. Third, we didn't do re-test reliability because online surveys couldn't guarantee that participants of the two surveys was the same person. In the future, a field survey can be conducted to verify test-retest reliability. Fourth, our study only investigates the effect of leadership attention on self-perceived continuous improvement in IPC from the perspective of organization incentives. Future research also needs to explore the relationship between self-perceived continuous improvement in IPC and the actual improvement. Fifth, our study was conducted during the pandemic. The level of leadership attention, incentives, and self-perceived continuous improvement in IPC may be overestimated, but the mechanism of leadership attention affecting self-perceived continuous improvement in IPC will not change because of these backgrounds.

## Conclusion

Leadership attention positively affects self-perceived continuous improvement in IPC, and incentives mediate the relationship between leadership attention and self-perceived continuous improvement in IPC. This finding offers recommendations that strengthening leadership construction and improving incentives systems in IPC are effective ways to enhance the effect of self-perceived continuous improvement in IPC.

## Data availability statement

The raw data supporting the conclusions of this article will be made available by the authors, without undue reservation.

## Ethics statement

The studies involving human participants were reviewed and approved by the Ethics Committee of Tongji Medical College, Huazhong University of Science and Technology (No: 2020S252). The patients/participants provided their written informed consent to participate in this study.

## Author contributions

LW conducted the literature review, took part in the investigation, contributed to the data cleaning, performed formal analysis, and wrote the original draft. DZ, JC, XL, and XZ designed the study, obtained funding, contributed to the interpretation of the results, and performed revisions of the manuscript. JL, YT, QZ, FZ, and QW contributed to the data cleaning and performed revisions of the manuscript. All authors contributed to the article and approved the submitted version.

## Appendix

**Leadership attention**

a1. Leaders in my unit support and actively participate in IPC tasks.
a2. Leaders in my unit supervise and check IPC tasks at any time.
a3. Leaders in my unit value suggestions on improving IPC work.

**Incentives in IPC**

a4. Evaluation results in IPC affect the income of staff in our unit.
a5. Staff in my unit are praised for pointing out hidden dangers or unsafe behaviors.
a6. Staff in my unit receive material rewards for better IPC performance.
a7. Apart from the material rewards, staff in my unit also get other rewards (e.g., promotion) for better IPC performance.

**Self-perceived continuous improvement in IPC**

a8. My unit regularly assess workflow to determine whether it needs improvement.
a9. My unit timely evaluate IPC measures to see how effective they are.
a10. My unit timely adjust workflow to ensure that the same HAIs do not happen again.
a11. Staff in my unit regularly receive feedback on the effect of continuous improvement.
